# Account of Diseases in an Eastern District of London, from June 20 to July 20, 1806

**Published:** 1806-08

**Authors:** 


### *Account of Diseases in an Eastern District of London, from June 20 to July 20, 1806.*

| | | | |
|---|---|---|---|
| ACUTE DISEASES. | | Amenorrhœa - - - | 7 |
| Rubeola - - - - - - | 3 | Fluor Albus - - - - - | 6 |
| Ophthalmia - - - - | 4 | Hypochondriasis - - | 3 |
| CHRONIC DISEASES. | | Hæmorrhois - - - | 2 |
| Tussis - - - - - | 8 | Rheumatismus Chronicus | 14 |
| Dyspnœa - - - - - | 9 | PUERPERAL DISEASES. | |
| Tussis cum Dyspnœa - | 7 | Ephemera - - - - | 4 |
| Phthisis Pulmonalis - | 4 | Mastodynia - - - - | 3 |
| Hæmoptysis - - - | 2 | Dolores Post Partum - | 2 |
| Cephalalgia - - - - | 4 | INFANTILE DISEASES. | |
| Gastrodynia - - - - | 6 | Erysipelas Infantilis - | 3 |
| Enterodynia - - - | 4 | Vermes - - - - - - | 4 |
| Anasarca - - - - - | 5 | Pertussis - - - - - - | 6 |
| Ascites - - - - - | 2 | Marasmus - - - - - | 4 |

*A List*

